# Reprogramming Photodynamic Therapy with BODIPY Photosensitizers: From Local Phototoxicity Toward Precision Photodynamic Oncology

**DOI:** 10.3390/molecules31183223

**Published:** 2026-09-12

**Authors:** Lina Deng, Yimiao Zhou, Zuowei Xiao

**Affiliations:** 1 Hunan Engineering and Technology Research Center for Health Products and Life Science, School of Pharmacy, Hunan University of Chinese Medicine, Changsha 410208, China; dln15273442653@126.com; 2 Xiangyin Campus, Xiangxing College, Hunan University of Chinese Medicine, Yueyang 414615, China; lauriechow@126.com; 3 Homologous Innovation Laboratory of Medicine and Food, Hunan University of Chinese Medicine, Changsha 410208, China

**Keywords:** photodynamic therapy, BODIPY photosensitizers, tumor microenvironment, tumor hypoxia, image-guided therapy

## Abstract

Photodynamic therapy (PDT) offers spatially confined, repeatable and mechanistically orthogonal tumor control, yet its broader clinical impact remains limited by inadequate light access, oxygen dependence and incomplete biological selectivity. Boron–dipyrromethene (BODIPY) dyes provide an unusually programmable platform for addressing these constraints. Although native BODIPYs are fluorescence-biased and inefficient triplet photosensitizers, their absorption, intersystem crossing, reactive oxygen species identity, aggregation, targeting and activation can be independently engineered through modular scaffold modification. In this Review, we present BODIPY as a programmable photochemical chassis for precision-oriented PDT. We first define the structure–photophysics–function relationships that convert BODIPY fluorophores into red- or near-infrared-responsive, type I- or type II-tunable and formulation-compatible photosensitizers. We then introduce a tumor-context-adaptive framework that integrates light-delivery routes, oxygen economy, multiscale targeting, organelle vulnerability and microenvironment-locked activation. Finally, we examine how BODIPY PDT can extend beyond direct cytotoxicity to vascular remodeling, immunogenic cell death, the potential to support in situ vaccination and rational combination therapy, while proposing translational go/no-go criteria for clinically credible candidates. We argue that progress will depend less on molecular complexity than on indication-first co-design of BODIPY chemistry, tumor biology and deployable light hardware.

## 1. Introduction

Cancer remains one of the defining biomedical challenges of this century. In 2024, approximately 20.6 million new cancer cases and 9.8 million cancer-related deaths occurred worldwide, and the annual number of new cases is projected to reach approximately 34.4 million by 2050 [1,2]. Although major advances in targeted therapy, immune checkpoint blockade, antibody–drug conjugates, and cell therapy have reshaped cancer treatment, durable local control and complete eradication of residual disease remain difficult in many clinical settings [3,4,5]. Recurrent superficial tumors, mucosal premalignant lesions, endoscopically accessible cancers, positive surgical margins, field cancerization and treatment-resistant local disease all illustrate a persistent therapeutic gap: the need for interventions that are spatially precise, repeatable, minimally invasive and biologically compatible with multimodal oncology [6,7,8].

Photodynamic therapy (PDT) is uniquely positioned within this gap [9]. Unlike systemic cytotoxic agents or molecular inhibitors that act continuously after administration, PDT is activated only when three components converge at the disease site: a photosensitizer, light of an appropriate wavelength and molecular oxygen [10,11]. Upon irradiation, the excited photosensitizer undergoes intersystem crossing to a triplet state and transfers energy or electrons to surrounding substrates, generating reactive oxygen species (ROS) [12]. These ROS can oxidize lipids, proteins, nucleic acids and organelle membranes, leading to tumor-cell death, vascular damage, stromal remodeling and, in some contexts, immune activation [13,14]. This externally controlled mechanism gives PDT an intrinsic spatial logic: drug distribution defines potential sensitivity, whereas light delivery defines the actual treatment field [15].

Porfimer-sodium-based PDT and 5-aminolevulinic-acid-derived protoporphyrin IX approaches have established the feasibility of light-activated cancer treatment in selected indications, including skin cancers, actinic keratosis, Barrett-associated dysplasia, obstructive esophageal cancer, early endobronchial lung cancer, bladder lesions and other anatomically accessible diseases [16,17]. PDT can also preserve tissue architecture, be repeated, and be combined with surgery, chemotherapy, radiotherapy or immunotherapy [18]. These properties remain attractive in modern oncology, especially for local disease control and residual microscopic disease [19]. Yet, despite decades of mechanistic insight and clinical experience, PDT has not become a broadly deployed therapeutic platform. The reasons are well known but often discussed separately [20]. First, light delivery imposes an anatomical constraint. Visible light can effectively treat superficial or cavity-accessible disease, but penetration through thick, pigmented or highly scattering tissues remains limited [21]. Red and near-infrared excitation, optical fibers, endoscopic systems, intraoperative illumination, two-photon excitation, upconversion and internal-light strategies have expanded the technical repertoire, but many claims of deep-tissue PDT remain difficult to translate into routine clinical practice [22]. Second, oxygen dependence limits classical Type II PDT. Many solid tumors contain chronic and cycling hypoxic regions, and PDT itself can aggravate oxygen depletion by consuming local oxygen during ROS generation [23]. Third, photosensitizer selectivity is uneven. Passive accumulation rarely guarantees sufficient tumor-to-normal contrast, and off-target retention can cause skin photosensitivity or damage to adjacent normal tissues [24]. These limitations indicate that the central challenge is not simply to create “stronger” photosensitizers but to design photosensitizers that are matched to disease anatomy, oxygen state, tumor biology and realistic light hardware [25].

Boron–dipyrromethene (BODIPY) dyes are unusually well suited to this redefinition of PDT [26]. The rigid dipyrromethene–BF_2_ core combines high molar absorptivity, photostability, sharp spectral profiles and exceptional synthetic tractability, whereas the meso (C8), α (C3/C5), β (C1/C2/C6/C7) and boron-center positions (B4), together with aza-BODIPY variants, provide orthogonal handles for tuning absorption, emission, intersystem crossing, ROS output, amphiphilicity, aggregation, subcellular trafficking and stimulus responsiveness [27,28,29]. Yet native BODIPY is not an intrinsically ideal photosensitizer: in most unsubstituted scaffolds, excited-state decay is dominated by fluorescence rather than productive triplet formation, resulting in limited intrinsic phototoxicity [30]. That paradox is precisely the attraction of the platform. BODIPY is valuable not because it is already optimal but because it is unusually amenable to converting photophysical liabilities into deliberate design variables [31].

Over the past decade, this design latitude has transformed BODIPY from a fluorescent label into a modular phototherapeutic scaffold [32]. π-Extension, aza substitution, donor–acceptor engineering, annulation, ring fusion and boron-center modification can shift absorption towards red and near-infrared windows, although often with trade-offs in triplet energetics, photostability or fluorescence brightness [33]. Triplet access can be amplified through heavy-atom substitution, spin–orbit charge-transfer states, orthogonal donor–acceptor geometries, metal coordination and heavy-atom-free strategies, enabling more nuanced control over Type II singlet-oxygen production versus Type I radical chemistry [34,35]. Equally important, supramolecular design has recast aggregation from a source of aggregation-caused quenching into a controllable determinant of pharmacology and photoactivity through amphiphilic programming, J-aggregation, aggregation-induced-emission-like behavior and nanoscale self-assembly [36]. This matters because the decisive limitation of PDT is no longer the absolute potency of an isolated photosensitizer. The constraining variables are contextual: anatomical access to light, intratumoral oxygen heterogeneity, tumor and organelle selectivity, and the need to integrate local oxidative injury with vascular, immune and systemic therapeutic responses [37]. A clinically credible photosensitizer must therefore be matched to disease site, illumination route, oxygen economy, cellular target and intended biological output. In this framework, BODIPY is not simply another organic photosensitizer class; it is a chemically programmable platform for co-optimizing imaging, activation logic, ROS identity and therapeutic combination within a single molecular architecture.

This Review therefore argues that the future of PDT should not be framed as a search for universally stronger photosensitizers. Here, precision photodynamic oncology denotes an indication-specific design framework in which photosensitizer chemistry, light delivery, tumor anatomy, oxygen state and biological vulnerability are matched to a defined indication; it is not used as an established clinical category. Rather, progress will depend on context-aware photosensitizers that are engineered around disease anatomy, microenvironmental oxygen status, tumor biology and deployable light hardware. From this perspective, BODIPY may help move PDT from a largely local phototoxic modality toward precision photodynamic oncology through image-guided, oxygen-state-aware and combination-ready design. We first examine why PDT remains clinically relevant yet translationally constrained. We then define the BODIPY scaffold as a privileged but incomplete photosensitizer platform, before discussing the design grammar that governs wavelength, triplet formation, ROS identity and aggregation. Finally, we integrate these principles with tumor context, light delivery, oxygen economy, multiscale selectivity, organelle vulnerability, immune remodeling and rational combination therapy and propose translational filters for clinically credible BODIPY systems. Our central premise is straightforward: the most important advance will be not more elaborate molecules per se but indication-first BODIPY systems in which chemistry, tumor biology and light hardware are deliberately co-designed.

**Figure 1:**
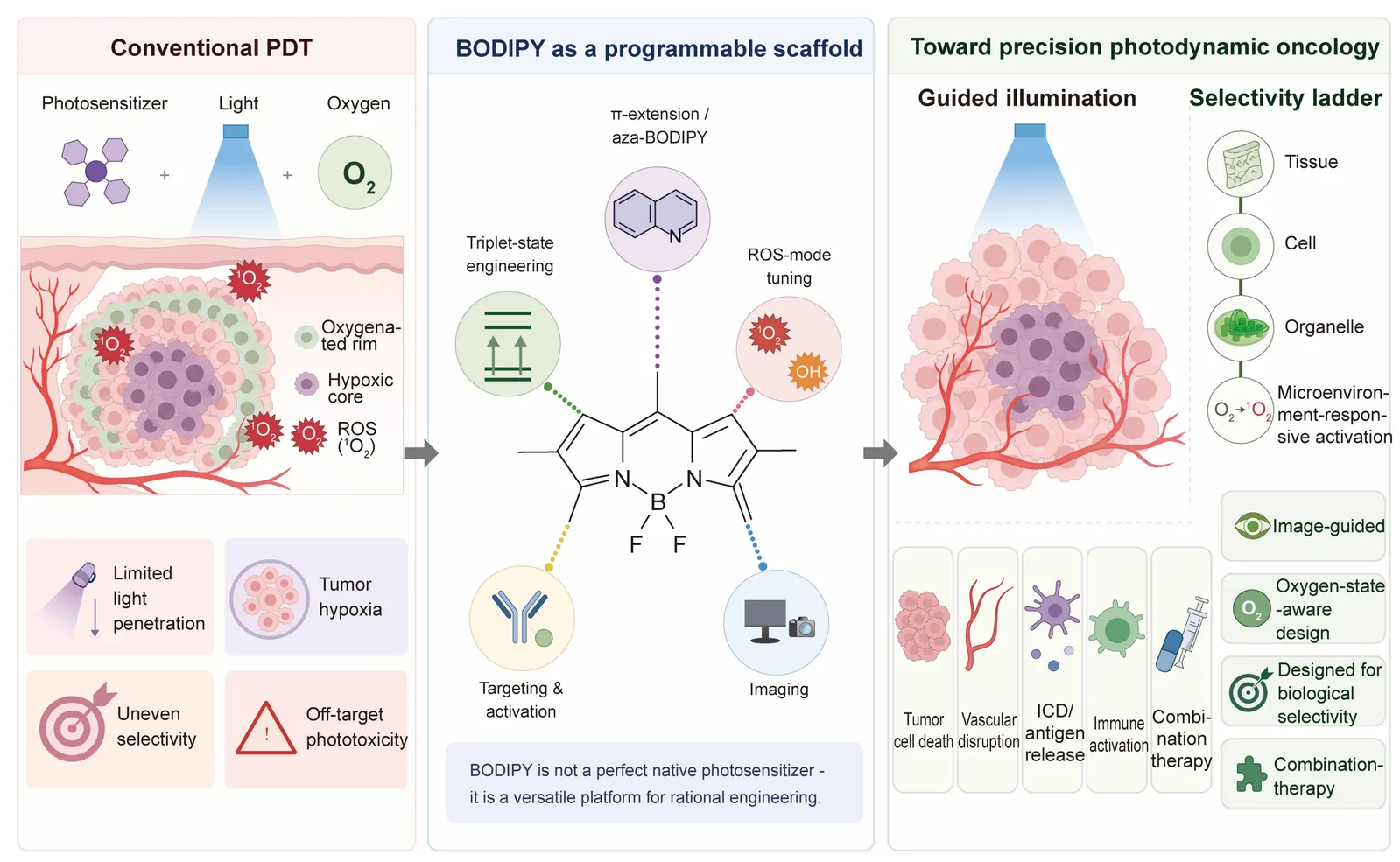
Reprogramming photodynamic therapy with BODIPY photosensitizers.

Conventional photodynamic therapy (PDT) requires the spatial convergence of a photosensitizer, activating light and molecular oxygen. Although this triad enables localized reactive oxygen species (ROS) generation, therapeutic efficacy is often limited by incomplete light penetration, heterogeneous tumor oxygenation, variable photosensitizer accumulation and off-target phototoxicity. The central panel illustrates BODIPY as a programmable, rather than intrinsically optimal, photosensitizer scaffold. Structural modification can extend absorption towards the red and near-infrared regions, enhance triplet-state formation, tune Type I versus Type II ROS production, introduce tumor-targeting or microenvironment-responsive functions, and preserve imaging capability. These properties are intended to support a transition toward precision photodynamic oncology, in which image-guided illumination is combined with a multiscale selectivity hierarchy encompassing tissue enrichment, tumor-cell recognition, organelle localization and microenvironment-responsive activation. Depending on photosensitizer localization, ROS identity and light dose, BODIPY PDT can induce direct tumor-cell killing, vascular disruption, immunogenic cell death and antigen release and can be integrated with systemic therapies. The figure therefore presents BODIPY PDT as a framework for image-guided, oxygen-state-aware and combination-ready development, in which biological selectivity must be demonstrated, rather than simply a more potent form of conventional PDT. BODIPY, boron–dipyrromethene; ICD, immunogenic cell death; NIR, near-infrared; PDT, photodynamic therapy; ROS, reactive oxygen species. This conceptual framework is illustrated in Figure 1.

## 2. Why Photodynamic Therapy Still Matters in Modern Oncology

### 2.1. Clinical Unmet Need and the Overlooked Value of PDT

Modern oncology has been transformed by molecularly targeted agents, immune checkpoint blockade, antibody–drug conjugates, cellular therapy and increasingly precise radiotherapy [38]. These advances have altered the natural history of several malignancies, yet they have not eliminated the need for locally controllable, organ-sparing and repeatable interventions. Many clinically important problems remain spatial rather than purely systemic: recurrent superficial tumors, mucosal premalignant disease, field cancerization, endoscopically accessible lesions, positive or close surgical margins, residual microscopic disease and local relapse after prior surgery or radiotherapy [7]. In these settings, the therapeutic objective is not always maximal systemic exposure but precise control of a defined anatomical field with limited collateral injury [39].

PDT is particularly relevant to this unmet need because it is governed by a distinct therapeutic logic. PDT requires the convergence of a photosensitizer, light of an appropriate wavelength and molecular oxygen. None of these components is sufficient alone [40]. Drug distribution defines the region that can potentially respond, whereas the illuminated volume defines where treatment is actually executed. This dual requirement gives PDT a level of spatiotemporal control that is difficult to achieve with conventional systemic drugs [41]. Upon irradiation, the excited photosensitizer transfers energy or electrons to surrounding substrates, generating ROS, including singlet oxygen and radical species. These short-lived oxidants damage lipids, proteins, nucleic acids and organelle membranes, producing tumor-cell death, vascular injury and, under appropriate conditions, immunogenic stress signaling [42].

This mechanism gives PDT three clinically relevant advantages. First, its activity can be anatomically restricted. For skin, oral, esophageal, airway, bladder and surgically exposed lesions, light can be delivered directly to the disease site, thereby limiting toxicity to the illuminated field. Second, its cytotoxic mechanism is orthogonal to most targeted agents. PDT kills through multitarget oxidative injury rather than inhibition of a single oncogenic driver, which may reduce cross-resistance with kinase inhibitors, endocrine therapy, chemotherapy or radiotherapy. Third, PDT can generate biological consequences beyond direct tumor-cell ablation. Vascular shutdown, stromal perturbation, antigen release and damage-associated molecular patterns can connect local oxidative injury to immune activation, providing a rationale for combining PDT with immunotherapy or other systemic modalities [43,44,45].

The overlooked value of PDT is therefore not that it can replace modern systemic oncology but that it can occupy a therapeutic niche that systemic agents often cannot fill [46]. It is most compelling in disease settings that are locally accessible, anatomically bounded, organ-preserving, repeat-treatment compatible and clinically dependent on durable local control [47]. This includes premalignant or early superficial disease, luminal lesions treatable by endoscopy, residual tumor in operative fields and selected cases in which local control may create a more favorable window for systemic therapy [48]. In this sense, PDT should not be evaluated as a generic treatment for all solid tumors. It should be developed as an indication-specific local modality whose value derives from spatial precision, mechanistic complementarity and biological compatibility with multimodal cancer care [49].

### 2.2. From Approved Local Therapy Toward a Context-Specific Platform

PDT is already an established clinical modality rather than an experimental concept. Approved and clinically adopted applications span a range of cancers and precancerous conditions, particularly in dermatology and endoscopically accessible disease [50]. These successes demonstrate the practical value of PDT as a localized, organ-preserving treatment [51]. However, PDT has yet to evolve into a broadly applicable precision oncology platform. Three challenges continue to limit its wider impact: light delivery, oxygen dependence and biological selectivity [52].

First, light penetration remains a fundamental constraint. PDT is most effective in superficial lesions, mucosal surfaces and sites accessible by endoscopy, optical fibers or surgery. Although advances such as near-infrared photosensitizers, interstitial illumination and internal light-generation strategies have expanded the treatment toolbox, reliable treatment of large or deeply seated tumors remains difficult, and many proposed solutions are still at the preclinical stage [53]. As a result, PDT should be viewed as a drug–device modality in which photosensitizer design and light-delivery strategy are inseparable. Second, most PDT mechanisms remain oxygen-dependent. Tumor hypoxia is common in solid cancers and can be further exacerbated during treatment as oxygen is consumed during ROS generation. Consequently, PDT efficacy often declines in the very tumor regions that are hardest to eradicate. This challenge has driven the development of oxygen-generating systems, Type I photosensitizers, metabolic interventions and hypoxia-responsive strategies [54]. Increasingly, the key question is not how much ROS a photosensitizer can generate under ideal conditions but how effectively it functions within the heterogeneous oxygen landscape of real tumors. Third, biological selectivity remains imperfect. Preferential tumor accumulation alone rarely provides sufficient discrimination between malignant and normal tissues [55]. Off-target retention can lead to photosensitivity and collateral damage, while tumor heterogeneity further limits the effectiveness of passive targeting. Improving the therapeutic index therefore requires multiple layers of selectivity, including tumor targeting, subcellular localization and disease-specific activation mechanisms [56].

Together, these challenges explain why PDT remains clinically valuable yet underutilized. They also define the priorities for next-generation photosensitizer development. Rather than pursuing universally more potent molecules, future designs should be tailored to specific clinical contexts, integrating realistic light-delivery routes, tumor oxygen status and biological vulnerabilities. This perspective naturally highlights programmable platforms such as BODIPY, which offer exceptional flexibility in tuning optical properties, ROS generation pathways, targeting capability and activatable behavior. Viewed through this lens, PDT is not an outdated local therapy but an underdeveloped context-specific modality whose full potential depends on better integration of chemistry, biology and device engineering.

## 3. Programming BODIPY Photophysics: Converting a Fluorescent Dye into a Programmable Photosensitizer

The photodynamic performance of BODIPY is governed by coupled structural, excited-state and biological variables. The upper panel maps the principal chemically addressable positions of the BODIPY scaffold, including the meso (C8), α (C3/C5), β (C1/C2/C6/C7) and boron-center (B4) positions [29]. π-Extension, ring fusion and aza substitution can narrow the electronic energy gap and shift absorption towards red or near-infrared wavelengths; donor–acceptor architectures and triplet-state engineering can promote intersystem crossing; and charge, amphiphilicity and self-assembly modules can regulate aqueous behavior, cellular uptake and formulation. The middle panel summarizes the resulting photophysical consequences. Structural modification alters the absorption window, the competition between fluorescence and triplet-state formation, and the balance between Type II energy transfer, which generates singlet oxygen, and Type I electron-transfer pathways, which generate radical species. Aggregation must also be controlled: disordered aggregation can cause aggregation-caused quenching, whereas programmed self-assembly or J-type aggregation can produce useful spectral shifts and preserve or enhance photodynamic function. These design axes are interdependent; for example, red-shifting absorption can improve tissue-compatible excitation but may reduce triplet energy, ROS yield or photostability. The lower panel links photophysical engineering to functional outcomes, including imaging guidance, performance across defined oxygen states, targeting selectivity, tumor-cell and vascular injury, and translational performance. ACQ, aggregation-caused quenching; BODIPY, boron–dipyrromethene; ISC, intersystem crossing; NIR, near-infrared; PDT, photodynamic therapy; PK/PD, pharmacokinetics and pharmacodynamics; ROS, reactive oxygen species; S_0_, ground singlet state; S_1_, first excited singlet state; T_1_, first excited triplet state.

### 3.1. BODIPY as a Programmable but Incomplete Photosensitizer Scaffold

**Figure 2:**
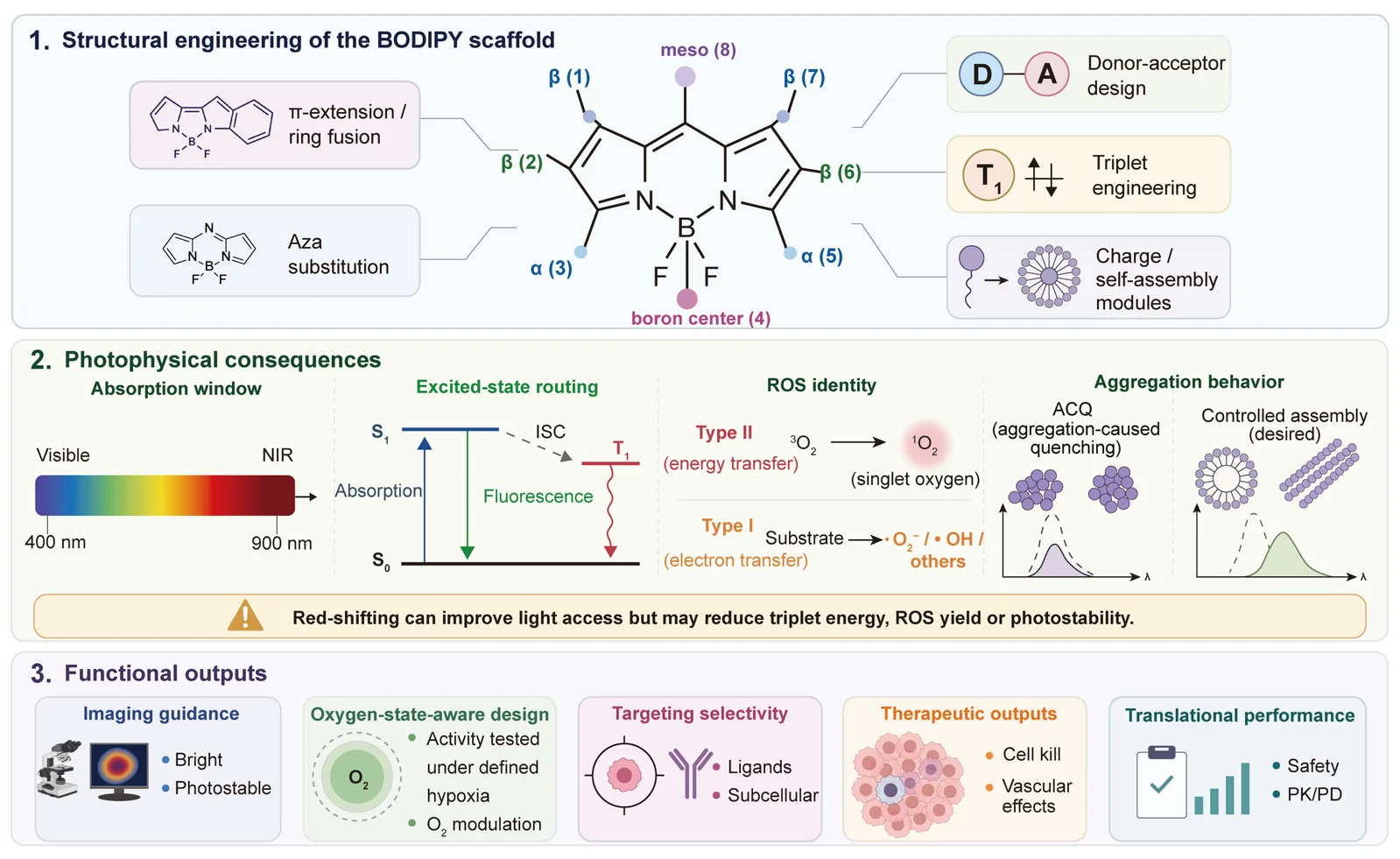
Structure–photophysics–function design grammar of BODIPY photosensitizers. Positional labels follow the Loudet–Burgess convention: C3/C5 are α, C1/C2/C6/C7 are β, C8 is meso and boron is B4 [29].

BODIPY dyes occupy a distinctive position in PDT: they are not naturally optimized photosensitizers but highly editable fluorophores that can be reprogrammed into therapeutic chromophores. The rigid dipyrromethene–BF_2_ core provides high molar absorptivity, narrow spectral bands, strong fluorescence, photostability and synthetic robustness, while the numbered meso, α, β and boron-center sites shown in Figure 2 allow modular modification of electronic structure, solubility, aggregation and biological behavior [27,29,57,58]. Aza-BODIPY variants further expand this design space by lowering the energy gap and shifting absorption into the red and NIR regions [59,60]. This structural editability distinguishes BODIPY from many classical photosensitizer families: rather than relying on a fixed porphyrinoid macrocycle or metal-centered excited state, BODIPY offers a compact scaffold in which optical output, triplet formation, ROS identity and targeting logic can be tuned in parallel [61].

The key limitation is equally important. Native BODIPY is usually a fluorescence-biased dye. Efficient radiative decay, small Stokes shifts and weak intersystem crossing (ISC) make many parent BODIPYs excellent imaging probes but poor ROS generators [30]. This photophysical bias explains why simple BODIPY fluorescence does not automatically translate into useful PDT. The field has therefore shifted from asking whether BODIPY is intrinsically phototoxic to asking how its excited-state landscape can be deliberately rerouted. In this sense, BODIPY should be viewed as a programmable photochemical chassis: fluorescence dominance, weak triplet access, visible-region absorption and aggregation sensitivity are not fixed defects but variables that can be engineered for a defined oncological context [32,62].

This reframing matters for context-specific PDT design. A photosensitizer for modern oncology must do more than generate ROS under ideal solution conditions. It must absorb light compatible with tissue and device constraints, generate the appropriate ROS under heterogeneous oxygen tension, remain sufficiently bright for image guidance when needed, localize to vulnerable cellular compartments and maintain photochemical function after formulation [63,64]. BODIPY is attractive because these properties can be co-designed rather than appended sequentially. The detailed design grammar is summarized in Figure 2. The class-level comparison of photosensitizer platforms is summarized in Table 1.

Porphyrins and chlorins retain stronger clinical PDT precedent, whereas phthalocyanines provide intense red/far-red absorption, cyanines offer mature NIR imaging capabilities, and AIE photosensitizers are designed to function in the aggregated state. The differentiating strength of BODIPY has therefore not demonstrated class-wide therapeutic superiority but structural programmability and the capacity to co-design optical response, ROS or thermal routing, imaging, targeting, activation and formulation for a defined indication.

### 3.2. Engineering BODIPY Absorption for Clinically Useful Light Windows

Most parent BODIPYs absorb in the green visible region, which is suboptimal for tissue illumination. Red and NIR excitation improve compatibility with clinical light delivery because longer wavelengths undergo less scattering and absorption by endogenous chromophores. BODIPY red-shifting has therefore become a central design objective. Major strategies include π-extension, styryl substitution, donor–acceptor construction, aza substitution, annulation, ring fusion and boron-center modification [68]. Each strategy narrows the HOMO–LUMO gap through a different mechanism: π-extension increases conjugation length, aza substitution stabilizes the LUMO, donor–acceptor motifs introduce intramolecular charge transfer, and ring fusion rigidifies the chromophore while extending delocalization [69,70].

However, red-shifting is not a sufficient design endpoint. The most common weakness of the field is to equate longer wavelength with better PDT. This is chemically and biologically incomplete. Excessive charge-transfer character can increase non-radiative decay; extended conjugation can reduce triplet-state energy; NIR emission may be gained at the expense of ROS yield; and hydrophobic red/NIR BODIPYs often require formulation strategies that alter their excited-state behavior [71]. Thus, clinically useful BODIPY photosensitizers must be optimized as balanced photochemical systems, not simply as long-wavelength dyes.

This balance is particularly important for cancer. A green-absorbing BODIPY with high singlet-oxygen yield may be effective in monolayer cells but poorly matched to tissue. Conversely, an NIR-absorbing BODIPY with weak triplet access may be optically attractive but therapeutically underpowered. The most relevant design space lies between these extremes: red/NIR absorption sufficient for the intended anatomical site, preserved triplet energetics, manageable photobleaching, adequate fluorescence or photoacoustic contrast when image guidance is required, and formulation stability under biological conditions [72]. BODIPY chemistry enables this tuning, but the optimal solution depends on indication and illumination route.

### 3.3. Engineering BODIPY Triplet States and ROS Identity

The therapeutic value of BODIPY PDT is determined by how efficiently absorbed energy reaches a productive ROS-generating state. Classical BODIPY triplet engineering relies on heavy-atom substitution, especially bromination or iodination at pyrrolic positions, which enhances spin–orbit coupling and promotes ISC [73]. This strategy can markedly increase singlet-oxygen production and has underpinned many early BODIPY photosensitizers. Yet it is not a universal solution. Heavy atoms may reduce fluorescence, increase dark toxicity, modify pharmacokinetics, promote photobleaching or complicate safety arguments for translational development [74]. The goal is therefore not merely to maximize ISC but to control triplet access without sacrificing biological deployability.

Heavy-atom-free strategies have become increasingly important. Orthogonal donor–acceptor architectures can promote spin–orbit charge-transfer ISC, enabling triplet formation through excited-state geometry and charge separation rather than halogenation [75]. Twisted donor–acceptor BODIPYs, dyads and charge-transfer systems can decouple fluorescence and triplet generation, permitting imaging and PDT functions to be balanced within the same scaffold [75]. Metal coordination and organometallic BODIPY dyads provide another route by combining the strong absorption of BODIPY with metal-centered or charge-transfer triplet states [76,77]. These approaches illustrate a broader principle: triplet formation is no longer simply a substituent effect but an excited-state design problem.

Equally important is the identity of ROS. Type II PDT, based on energy transfer to molecular oxygen and generation of singlet oxygen, remains central to many BODIPY systems. However, solid tumors are spatially heterogeneous in oxygenation, and PDT itself can further deplete oxygen. This makes exclusive reliance on singlet oxygen biologically fragile [11,34]. Type I pathways, in which electron or hydrogen transfer generates superoxide, hydroxyl radicals and related radical species, are increasingly attractive because they may retain activity under lower oxygen tension or exploit redox-rich tumor microenvironments [78]. BODIPY scaffolds can be biased towards Type I or Type II behavior by tuning redox potential, donor–acceptor strength, charge-transfer character, aggregation state and local microenvironment [79].

This shift has practical consequences. Singlet-oxygen quantum yield alone is no longer an adequate ranking metric. A BODIPY candidate should be evaluated by ROS identity, oxygen dependence, photostability, subcellular localization and biological outcome under relevant oxygen levels. For context-specific PDT, the most useful candidates may not be purely Type I or Type II. BODIPY systems designed for oxygen-state adaptation aim to combine Type II activity in oxygenated regions with Type I or redox-coupled activity at lower oxygen levels, but this remains a design objective that requires testing across defined oxygen conditions.

### 3.4. Engineering BODIPY Aggregation Rather than Merely Avoiding It

Aggregation is often treated as a formulation problem, but for BODIPY it is a central photophysical variable. Many hydrophobic BODIPYs self-associate in aqueous media, and uncontrolled H-type aggregation can suppress fluorescence and ROS generation through aggregation-caused quenching (ACQ) [80]. Early strategies focused on preventing this through PEGylation, ionic groups, surfactants, polymer carriers or nanoparticle encapsulation. These approaches remain useful, especially when monomer-like photophysics must be preserved. However, they do not fully exploit the opportunities offered by controlled assembly.

A more advanced view distinguishes unwanted aggregation from programmed supramolecular organization. J-aggregation, amphiphilic self-assembly, protein confinement, polymeric nanostructures and kinetically controlled aggregates can generate new optical states, alter exciton coupling, red-shift absorption, improve tumor retention and enable self-reporting theranostic behaviors [81]. In some systems, restricted intramolecular motion or aggregation-induced-emission-like behaviors restore fluorescence and photodynamic output in the assembled state [82]. In others, assembly changes the balance between fluorescence, heat generation and ROS production, enabling combined PDT, photothermal therapy and imaging [83].

This has direct translational relevance. A BODIPY that performs well as a monomer in organic solvent may fail after biological formulation if its aggregate state is photochemically inactive [84]. Conversely, a nanostructured BODIPY with moderate molecular ROS yield may outperform a highly active monomer in vivo because it has better stability, tumor retention, imaging feedback or microenvironment-triggered activation. Therefore, aggregation state should be characterized as part of the photosensitizer itself. Absorption shifts, fluorescence lifetime, ROS output, photobleaching, particle stability and biological activity should be evaluated in the final formulation, not inferred from dilute-solution measurements.

### 3.5. Design Principle

The design logic emerging from BODIPY PDT is integration. Wavelength, fluorescence, ISC, triplet lifetime, ROS identity, aggregation state, solubility, charge, localization and activatability are coupled variables. π-Extension may improve light access but weaken triplet energetics. Heavy atoms may improve ISC but reduce imaging value or increase toxicity. Donor–acceptor construction may enable heavy-atom-free triplets but increase environmental sensitivity. Amphiphilic assembly may improve pharmacology while reshaping fluorescence and ROS output. These trade-offs are not minor technical details; they define whether a BODIPY system is clinically meaningful. Next-generation BODIPY photosensitizers should therefore be developed as modular photochemical circuits rather than single-parameter optimized dyes. A candidate intended for superficial lesions may prioritize high ROS yield and simple red-light activation; a candidate for image-guided surgery may prioritize fluorescence contrast and activatable phototoxicity; a candidate for hypoxic tumor may require Type I bias, oxygen economy or hypoxia-triggered combination logic; and a candidate for systemic delivery must balance assembly, pharmacokinetics and safety. The important question is not whether BODIPY is the strongest photosensitizer class in general but whether BODIPY chemistry can be programmed to fit a specific tumor context. This is the central value of the scaffold. BODIPY converts photosensitizer development from empirical dye screening into rational co-design of optical physics, excited-state routing, supramolecular state and biological function. That is what allows a bright fluorophore to become a programmable photosensitizer.

Representative systems illustrate the breadth of BODIPY design strategies, from direct heavy-atom sensitization and heavy-atom-free charge-transfer engineering to activatable, organelle-targeted and supramolecular PDT/PTT platforms (Table 2). The maturity of the evidence is nevertheless uneven: quantitative ROS or phototoxicity measurements are not consistently accompanied by in vivo validation, and full pharmacokinetic, clearance, biodistribution and long-term safety datasets are frequently absent. Cell-only systems and in vivo formulations should therefore be interpreted as different evidence levels rather than equivalent demonstrations of translational readiness.

## 4. Deploying BODIPY as Tumor-Context-Adaptive Therapeutic Systems

**Figure 3:**
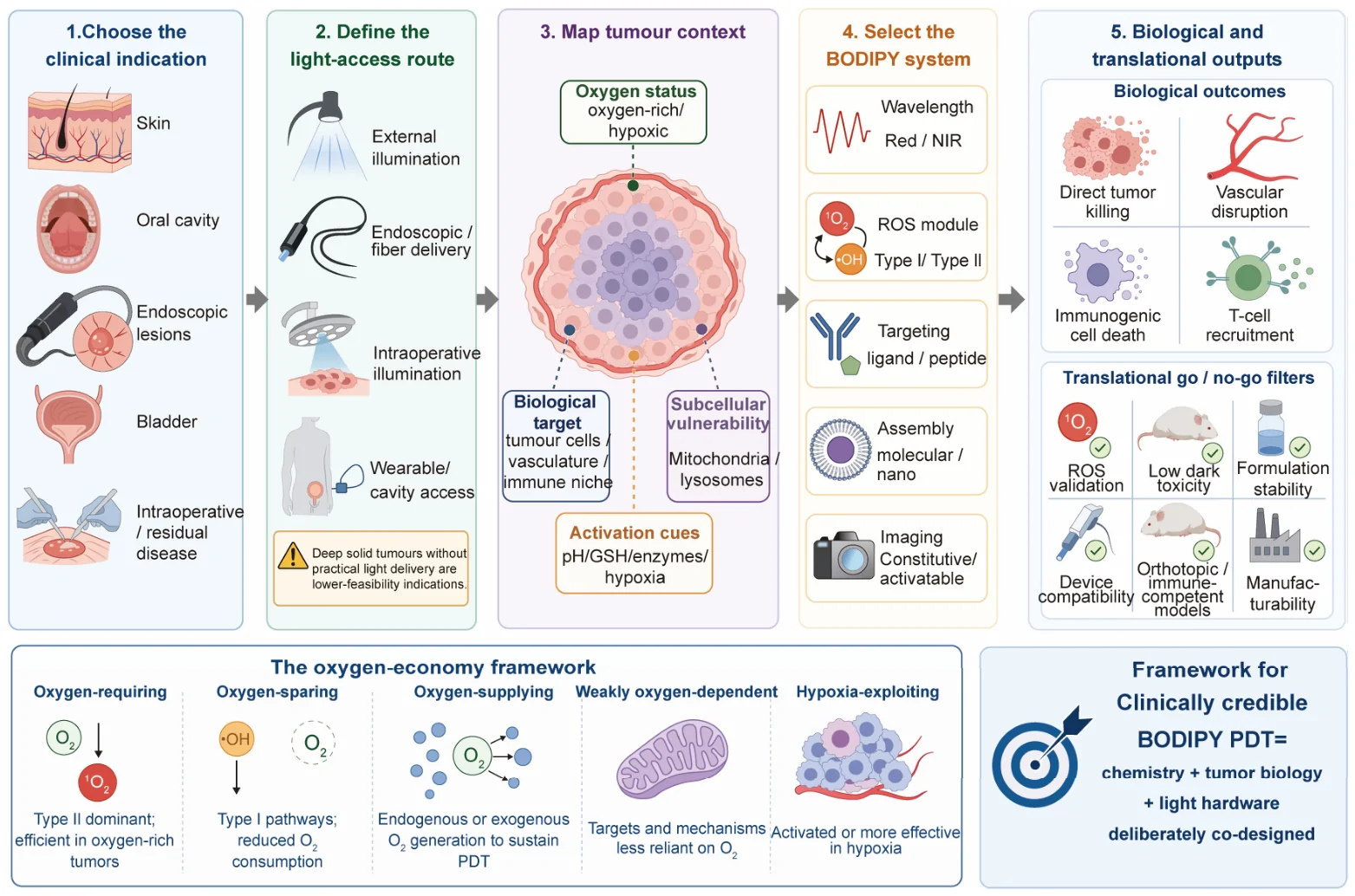
Tumor-context-adaptive and translational roadmap for BODIPY photodynamic therapy.

A clinically credible BODIPY PDT program should begin with the disease indication rather than with the photosensitizer molecule. Anatomically accessible settings—including skin and oral lesions, endoscopic disease, bladder tumors, intraoperative margins and residual local disease—provide the most direct opportunities for external, endoscopic, fiber-based, intraoperative or wearable illumination. Deep solid tumors without a practical light-delivery route remain less feasible, irrespective of photosensitizer potency. Once light access has been defined, the tumor context should be mapped, including oxygenation, the intended cellular or vascular target, subcellular vulnerabilities and activation cues such as acidity, glutathione, enzymes or hypoxia. These features guide selection of modular BODIPY properties, including wavelength, ROS mode, targeting ligand, molecular or nanoscale assembly and imaging readout. Biological outcomes may include direct tumor-cell killing, vascular disruption, immunogenic cell death and T-cell recruitment, but these claims require appropriate mechanistic validation. Translational progression should therefore be governed by explicit go/no-go criteria, including validated ROS identity, low dark toxicity, formulation stability, device compatibility, testing in orthotopic or immune-competent models and manufacturability. The lower panel presents an oxygen-economy framework that distinguishes oxygen-requiring, oxygen-sparing, oxygen-supplying, weakly oxygen-dependent and hypoxia-exploiting strategies. Not all modules need to be incorporated into one system; rather, BODIPY chemistry, tumor biology and light hardware should be deliberately co-designed for a defined clinical indication. BODIPY, boron–dipyrromethene; ER, endoplasmic reticulum; GSH, glutathione; NIR, near-infrared; PDT, photodynamic therapy; ROS, reactive oxygen species. Figure 3 outlines a tumor-context-adaptive and translational roadmap for BODIPY PDT.

### 4.1. BODIPY and the Light-Delivery Problem

For BODIPY photodynamic therapy (PDT), light delivery is not a secondary technical detail but a determinant of whether a molecule can become a therapy. The photochemical performance of a photosensitizer is meaningful only if its absorption spectrum can be matched to a clinically deliverable light field. This makes PDT a drug–device modality: the photosensitizer, anatomical site, illumination geometry, optical dose and treatment workflow must be designed together [90,91].

BODIPY chemistry offers several routes to improve optical compatibility. π-Extension, aza-BODIPY construction, donor–acceptor engineering, annulation and ring fusion can move absorption from the green visible region towards red and near-infrared wavelengths [92,93]. This is important because red and NIR light generally penetrate tissue more effectively than shorter-wavelength visible light. However, the translational meaning of “deep” PDT is often overstated. Longer wavelength improves optical access, but it does not abolish scattering, absorption, heterogeneous perfusion or tumor geometry. Clinically credible deployment still favors anatomically accessible indications: superficial skin lesions, oral and mucosal disease, bladder lesions, endoscopically accessible gastrointestinal or airway tumor, intraoperative tumor beds and residual local disease [94].

Several light-delivery strategies are relevant for BODIPY systems. External LED, laser or OLED sources are most plausible for skin, oral and wound-adjacent applications. Endoscopic and fiber-based illumination remain the most clinically realistic routes for luminal disease [95]. Intraoperative illumination may be particularly attractive for BODIPY photosensitizers that combine fluorescence-guided surgery with margin-directed PDT. Interstitial fiber diffusers can extend treatment to selected solid lesions but require careful dosimetry and image-guided placement. By contrast, two-photon excitation, upconversion nanoparticles, chemiluminescence, Cerenkov radiation and other “internal light” strategies are conceptually powerful but remain largely preclinical for oncology [96]. They should be framed as experimental solutions to optical access, not as immediate replacements for fiber, endoscopic or intraoperative light delivery.

This distinction matters for BODIPY development. A photosensitizer intended for a topical, endoscopic or intraoperative indication may not need NIR-II absorption if red-light activation, high photostability, shallow-field precision and simple formulation are sufficient [97]. Conversely, a molecule optimized for long-wavelength absorption may be therapeutically weak if triplet formation, ROS yield or formulation stability are compromised. The most rigorous route is therefore indication-first: define the clinical site and light hardware before selecting the BODIPY scaffold. In this framework, BODIPY photophysics should be tuned not for the longest possible wavelength but for the most useful balance between optical access, ROS generation, imaging contrast and deliverable light dose [98].

### 4.2. BODIPY and the Oxygen-Economy Problem

The second contextual constraint is oxygen. Classical Type II PDT depends on energy transfer from the triplet photosensitizer to molecular oxygen to generate singlet oxygen. This mechanism is efficient in oxygenated environments but fragile in solid tumors, where oxygen tension varies across vessels, necrotic regions, stromal barriers and cycling perfusion states [99]. PDT can intensify the problem by consuming oxygen during treatment, generating a negative feedback loop in which initial ROS production worsens local hypoxia and reduces subsequent photodynamic efficacy [100].

BODIPY systems can address this problem if they are designed around oxygen economy rather than simply singlet-oxygen yield. Oxygen-economy design includes five non-exclusive strategies. First, oxygen-requiring BODIPYs can be optimized for indications where oxygen supply is relatively favorable, such as superficial or well-perfused lesions. Second, oxygen-sparing systems can reduce local oxygen consumption, for example, by combining PDT with mitochondrial respiration inhibition or metabolic modulation [101]. Third, oxygen-supplying systems can incorporate perfluorocarbon domains, catalase-like components, MnO_2_-based nanomaterials or hemoglobin-inspired carriers to increase local oxygen availability [101,102]. Fourth, weakly oxygen-dependent or Type-I-biased BODIPYs can use electron-transfer chemistry to generate radical species under lower oxygen tension [103]. Fifth, hypoxia-exploiting systems can intentionally couple PDT-induced oxygen depletion to hypoxia-activated prodrugs or HIF-related vulnerability [72,85].

BODIPY is well suited to this framework because its redox potential, charge-transfer character, aggregation state and subcellular localization can be tuned. Electron-rich donor–acceptor designs, heavy-atom-free triplet systems and charge-transfer BODIPYs can promote Type I pathways, whereas halogenated or otherwise triplet-enhanced BODIPYs may favor type II singlet oxygen under non-monic conditions [73]. The relevant metric is therefore not a single quantum yield measured in aerated organic solvent. A serious BODIPY candidate should be evaluated under biologically relevant oxygen levels, with explicit identification of ROS species, photobleaching, oxygen consumption and cell death outcome [86].

This shift also changes how hypoxia should be interpreted. Hypoxia is not merely an obstacle to be reversed. It is a spatially patterned tumor property that can be used to guide therapeutic design. For example, an oxygen-supplying BODIPY nanoassembly may be appropriate for a hypoxic but vascularized tumor margin, whereas a Type I or hypoxia-activated combination strategy may be better suited to poorly perfused regions. The future is therefore not “Type II versus Type I” as a binary choice, but BODIPY systems that can remain functional across oxygen gradients [104,105].

### 4.3. Oxygen-State-Adaptive BODIPY Photosensitizers

An oxygen-state-adaptive BODIPY photosensitizer would not rely on a single fixed photochemical output. Instead, it would be designed to operate differently across the oxygen heterogeneity of a tumor. In oxygenated regions, triplet BODIPY could generate singlet oxygen through Type II photochemistry. In intermediate oxygen regions, mixed Type I/Type II pathways may dominate. In severely hypoxic regions, radical generation, redox cycling, oxygen-sparing metabolism or hypoxia-activated drug release could provide compensatory activity [106].

This concept is still more of a design direction than a mature clinical class, but it is scientifically grounded. Organic photosensitizers can be engineered to favor Type I chemistry by tuning excited-state redox potential, intramolecular charge transfer and donor–acceptor geometry. Activatable photosensitizers can be built so that ROS production is switched on by tumor-associated triggers such as acidity, glutathione, enzymes, ROS/RNS or hypoxia [87]. BODIPY scaffolds can integrate these features with fluorescence or photoacoustic readouts, allowing oxygen-state adaptation to be coupled to imaging feedback [107].

The central challenge is avoiding unnecessary platform complexity. A BODIPY system designed for oxygen-state adaptation should not simply combine multiple components because each is individually attractive. Its modules should have a mechanistic sequence: light absorption, triplet access, oxygen-aware ROS generation, tumor-associated activation and biologically meaningful injury. For example, a BODIPY nanostructure could combine red-light absorption, Type-I-biased radical generation and hypoxia-activated chemotherapy only if each component solves a specific limitation of the same tumor setting. Otherwise, multifunctionality risks becoming a barrier to reproducibility, pharmacokinetics, manufacturing and regulatory translation [88]. For this reason, oxygen-state-adaptive PDT should be judged by stringent evidence. Studies should report ROS identity under normoxia and hypoxia, oxygen consumption during irradiation, maintenance of phototoxicity under low oxygen, mechanism of cell death, biodistribution and the contribution of each module. Without such evidence, “hypoxia-tolerant” or “oxygen-independent” claims remain weak. The BODIPY scaffold may enable oxygen-state adaptive design, but only if photochemistry, formulation and tumor biology are evaluated together.

### 4.4. BODIPY-Enabled Tumor Selectivity: Delivery Is Not Activation

Claims of tumor specificity in PDT are frequently oversimplified. Accumulation in tumor tissue is not equivalent to therapeutic selectivity. A photosensitizer may enter a tumor but remain inactive, localize to resistant compartments or damage adjacent normal tissue when illuminated. Conversely, a highly activatable photosensitizer may still fail if it does not reach the correct tissue or cell type. For BODIPY PDT, selectivity should therefore be separated into two questions: where does the photosensitizer go, and where does it become photodynamically active?

BODIPY can be engineered at both levels. Tissue and cell-level enrichment can be improved through passive nanocarrier accumulation, local administration, receptor ligands, peptides, antibodies, sugars, folate, biotin, membrane-affinity tuning and charge engineering [108]. Aza-BODIPY and other hydrophobic red/NIR derivatives can be formulated into micelles, polymeric nanoparticles, albumin complexes or supramolecular assemblies to improve circulation, retention and tumor penetration [71,109]. Cationic or amphiphilic modifications can increase membrane association and cellular uptake, whereas fluorination or hydrophobic tuning may alter plasma membrane affinity and subcellular trafficking [110]. Fluorescently labeled glycans provide tools for studying glycan-associated biological processes, but this fluorescence-labeling literature does not itself establish tumor targeting or PDT efficacy [111].

Activation-level selectivity is a different design problem. Activatable BODIPY systems can suppress fluorescence or ROS generation until they encounter tumor-associated conditions. Common triggers include acidic pH, high glutathione, tumor-associated enzymes, hypoxia, ROS/RNS, viscosity and polarity [112]. This logic is particularly important for PDT because off-target accumulation is less harmful if photosensitization remains caged until irradiation and selective activation in tumor tissue occur. However, trigger specificity must be evaluated carefully. Many “tumor microenvironment” cues are not unique to malignant tissue; acidity, redox changes and inflammation can occur in normal physiology or non-malignant disease. A robust BODIPY system therefore requires both delivery contrast and activation contrast. The most defensible selectivity model is layered. Tissue enrichment determines where BODIPY can accumulate. Cell recognition determines which tumor or stromal populations are preferentially reached. Organelle localization determines the vulnerability exposed to ROS. Microenvironment-responsive activation determines when phototoxicity is switched on. Each layer can fail independently.

### 4.5. Multiscale BODIPY Targeting

Subcellular localization is especially important because ROS are short-lived and act near their site of generation. Mitochondrial BODIPY photosensitizers can trigger membrane potential collapse, cytochrome c release, metabolic stress and apoptosis-like death. Lysosome-localized BODIPY can induce lysosomal membrane permeabilization, cathepsin release and downstream cell death. ER-localized PDT can promote ER stress and may favor immunogenic cell death when damage-associated molecular patterns are properly engaged. Plasma membrane-targeted BODIPY can produce rapid oxidative membrane injury, whereas lipid droplet or lipid-rich compartment targeting may enhance lipid peroxidation and ferroptosis-like death [113,114].

The BODIPY FL-labeled monoterpenoid reported by Guseva et al. was used for bio-visualization of bacterial and fungal cells and showed moderate mitochondrial tropism in mammalian cells [115]. It illustrates how BODIPY functionalization can alter biological localization, but it does not provide direct evidence of antitumor PDT efficacy.

BODIPY is suitable for this multiscale targeting because subtle structural changes can strongly alter biological distribution. Triphenylphosphonium, cationic pyridinium groups, morpholine, peptides, lipid chains, fluorination and amphiphilic balance can shift BODIPY between mitochondria, lysosomes, membranes or lipid compartments [116]. These changes affect not only where BODIPY localizes but also which ROS are generated, how far they diffuse, and which death program dominates. Thus, organelle targeting should not be treated as a decorative label. It is a functional determinant of therapeutic biology. Multiscale targeting also offers a way to integrate imaging and therapy. Fluorescent BODIPY can report accumulation, activation or organelle localization before irradiation, potentially allowing treatment fields or light doses to be adjusted. Activatable fluorescence–PDT systems are particularly attractive because they connect diagnosis, dosimetry and phototoxicity. However, imaging brightness and phototoxicity are often in tension: strong fluorescence can compete with triplet formation, whereas high ISC can quench fluorescence. The best BODIPY systems will not maximize both indiscriminately but will balance them according to clinical use. Image-guided surgery may prioritize pre-irradiation contrast and margin mapping, whereas ablation of a known superficial lesion may prioritize ROS output and formulation simplicity.

These design layers also introduce distinct failure modes. Receptor-directed delivery can be limited by inter- and intratumor receptor heterogeneity, receptor expression in normal tissues and poor tumor penetration even when ligand affinity is high [36]. Ligand conjugation and nanoscale formulation can alter biodistribution and circulation, while off-target uptake or clearance remain construct-dependent [36,108,109,110]. For organelle targeting, fluorescence co-localization alone does not demonstrate functional delivery because apparent localization is dynamic, varies with cell type and state, and may reflect non-specific membrane binding by cationic or lipophilic motifs. Mechanistic assignment should therefore couple co-localization with functional perturbation of the proposed organelle and evidence that localization changes the phototoxic response [110,113,114,116]. Microenvironment-responsive activation faces a different problem: pH, GSH, ROS and hypoxia form continuous spatial gradients rather than tumor-exclusive binary signals. Activation threshold, residual off-state leakage, activation kinetics and intratumor heterogeneity can therefore determine whether useful contrast is achieved [31,112]. Responsive constructs should be compared with appropriate non-responsive or constitutively active controls to show improvement in therapeutic index, not merely a fluorescence change. Across all three levels, targeting or activation should be regarded as functionally meaningful only when it measurably improves therapeutic selectivity or therapeutic index or produces a mechanistically relevant change in biological response, rather than merely changing fluorescence localization.

### 4.6. BODIPY-Triggered Oxidative Cell Death Programs

Once BODIPY reaches the correct tumor context, its biological output depends on ROS identity, localization, dose rate and cellular stress state. PDT can induce apoptosis, necrosis, autophagy-associated death, paraptosis-like injury, ferroptosis-like lipid peroxidation and other oxidative death programs [117]. These categories should not be treated as mutually exclusive. In many tumor models, PDT triggers overlapping damage cascades, with the dominant phenotype determined by organelle injury, antioxidant capacity, membrane composition and repair competence.

Mitochondria-targeted BODIPY often favors apoptotic or metabolic collapse phenotypes because mitochondrial membranes, electron transport and calcium homeostasis are directly vulnerable to oxidative injury [118]. Lysosomal BODIPY can promote protease release and lysosome-dependent death. ER-associated BODIPY may engage unfolded protein response pathways and expose calreticulin, potentially linking cell death to immunogenic signaling. Plasma membrane-localized photosensitizers can produce rapid loss of membrane integrity through contact-dependent lipid oxidation. Lipid-droplet-associated or membrane-proximal PDT can enhance lipid peroxidation, a process mechanistically related to but not identical to canonical ferroptosis [119].

This distinction is important. PDT-induced lipid peroxidation can resemble ferroptosis but may not always depend on the canonical enzymatic machinery of ferroptosis. The term “ferroptosis-like” should therefore be used carefully unless iron dependence, GPX4 axis involvement, rescue by ferroptosis inhibitors and lipid peroxidation markers are demonstrated. Similarly, claims of immunogenic cell death require evidence beyond cell death itself, including calreticulin exposure, ATP release, HMGB1 release, dendritic-cell activation or T-cell priming [37]. For BODIPY PDT, the most rigorous studies will connect photochemical measurements to cell-biological outcomes rather than using generic viability assays alone.

### 4.7. BODIPY PDT and Tumor Microenvironment Remodeling

The value of PDT in oncology is not limited to tumor-cell killing. PDT can damage tumor vasculature, alter perfusion, remodel stromal barriers, generate inflammatory signals and release tumor antigens [6]. These effects are context-dependent. Vascular-targeted PDT can produce vessel shutdown and rapid tumor ischemia, whereas cell-targeted PDT may primarily induce tumor-cell stress and antigen release. In either case, local oxidative injury can reshape the tumor microenvironment (TME).

BODIPY systems provide new opportunities to tune this remodeling. Image-guided BODIPY PDT can confine oxidative injury to tumor margins or residual lesions. Organelle-targeted BODIPY can bias the type of stress signal released. BODIPY systems designed for oxygen-state adaptation aim to maintain photodynamic or other photoactivated functions across hypoxic and oxygenated compartments. Combination-ready BODIPY systems can be paired with immune checkpoint blockade, photothermal therapy, ferroptosis induction, chemotherapy, hypoxia-activated prodrugs or radiotherapy, provided the combination has a clear mechanistic rationale [120].

The immune dimension is particularly important. PDT can promote immunogenic cell death, dendritic-cell maturation, antigen presentation and T-cell infiltration, but these outcomes are not automatic [121]. They depend on tumor immunogenicity, photosensitizer localization, treatment dose, timing, inflammatory context and host immune competence. Therefore, BODIPY PDT should not be described as immunotherapy unless immune mechanisms are directly shown. The strongest claim is more precise: appropriately designed BODIPY PDT can convert local oxidative injury into a microenvironmental perturbation that may support systemic immune therapy.

## 5. Future Directions of New BODIPY Photosensitizer-Based PDT

### 5.1. Biological Outputs Beyond Direct Cytotoxicity

The next stage of BODIPY PDT should be framed less as an exercise in maximizing ROS and more as a problem of engineering tumor biology. PDT has classically been described as a local phototoxic treatment in which light-activated photosensitizers generate short-lived ROS that kill cancer cells. This view is chemically correct but biologically incomplete. In tumor, photodynamic injury is filtered through subcellular localization, oxygen tension, antioxidant capacity, membrane composition, vascular state and immune context. The same amount of ROS can therefore produce different biological outputs depending on where and how it is generated [54,122].

BODIPY photosensitizers are well suited to exploit this principle because their structure can be tuned to control not only absorption and ROS mode but also cell uptake, organelle localization and activation state [123]. Mitochondria-targeted BODIPY systems can induce mitochondrial depolarization, respiratory failure, calcium imbalance and apoptosis-associated phenotypes. Lysosome-localized photosensitizers can promote lysosomal membrane permeabilization and protease release. Endoplasmic reticulum (ER)-associated PDT can engage ER stress, unfolded protein response pathways and immunogenic signaling. Plasma-membrane or lipid-compartment-localized photosensitizers can rapidly oxidize unsaturated lipids and disrupt membrane integrity [124]. Thus, BODIPY PDT should not be evaluated only by bulk cell viability; the relevant question is which cellular vulnerability is exposed by the photosensitizer’s localization and ROS chemistry.

This distinction is especially important for ferroptosis-like lipid peroxidation. Canonical ferroptosis is an iron-dependent, lipid-peroxidation-driven form of regulated cell death controlled by pathways such as cystine uptake, glutathione metabolism and GPX4 activity. PDT can generate extensive lipid peroxidation, and a distinct ferroptosis-like death program initiated by non-enzymatic photodynamic lipid oxidation has been described [125]. However, not every lipid peroxidation phenotype induced by PDT should be labeled ferroptosis. Claims of ferroptosis require evidence of iron dependence, lipid peroxide accumulation, sensitivity to ferroptosis inhibitors, and engagement of relevant antioxidant pathways. For BODIPY systems, rigorous mechanistic annotation will be essential: apoptosis, necrosis, autophagy-associated death, ferroptosis-like lipid peroxidation and other oxidative death programs should be distinguished experimentally rather than inferred from morphology or viability assays alone [126].

Beyond tumor-cell death, PDT can damage tumor vasculature, alter perfusion, expose tumor antigens, remodel stromal barriers and generate damage-associated molecular patterns (DAMPs) [127]. These effects are not secondary details; they determine whether local phototoxicity remains a purely ablative event or becomes a broader tumor-microenvironment perturbation. Future BODIPY photosensitizers should therefore be designed and tested according to biological output: whether they primarily debulk tumor cells, shut down vasculature, trigger lipid oxidative catastrophe, induce ER stress, promote antigen release or support immune priming.

### 5.2. Potential of BODIPY PDT to Support in Situ Vaccination

BODIPY PDT has the potential to support in situ vaccination only when local oxidative injury produces demonstrated immunogenic cell death (ICD), antigen presentation and T-cell priming; evidence of systemic tumor control or immune memory provides a stronger validation tier. ICD is characterized by coordinated emission of immunostimulatory signals, including calreticulin exposure, ATP secretion and HMGB1 release, which promote antigen uptake, dendritic-cell activation and T-cell priming [128]. This outcome is not automatic and depends on the photosensitizer, subcellular target, light dose, tumor genotype, immune competence and timing of treatment relative to immune modulation [129,130].

BODIPY can contribute to this field in several ways. First, its fluorescence or photoacoustic signal can support image-guided localization and controlled irradiation, reduce random tissue injury and improve the reproducibility of immune priming. Second, organelle-targeted BODIPY systems may influence the pattern of immunogenic stress, but such claims should be linked to established ICD readouts such as calreticulin exposure, ATP secretion and HMGB1 release [128,129,130]. Third, BODIPY platforms can be coupled with immunomodulators or nanoadjuvants for spatially controlled immune activation, provided that each component has a defined mechanistic role [131,132].

The immunological ambition should be precise. BODIPY PDT should not be described as immunotherapy unless immune mechanisms are directly demonstrated. Evidence should include DAMP release, dendritic-cell maturation, antigen presentation, CD8+ T-cell infiltration, memory formation or response to immune depletion studies. Synergy with immune checkpoint blockade should be claimed only when PDT increases tumor immunogenicity or T-cell responsiveness in immune-competent models [88]. The most valuable BODIPY systems will therefore be those that combine local control with measurable immune amplification: killing the illuminated lesion while increasing the probability that non-illuminated tumor deposits or residual microscopic disease become visible to the immune system [132].

### 5.3. Rational Combination Therapy

Combination therapy is a natural direction for BODIPY PDT, but it is also a common source of over-engineering. BODIPY platforms can be paired with photothermal therapy, chemotherapy, photoactivatable prodrugs, ferroptosis induction, radiotherapy, hypoxia-activated chemotherapy, metabolic inhibitors and immune checkpoint blockade [31,32,33,34,35,36,37,38,39]. Yet adding more functions to a nanoparticle does not automatically create a better therapy. A rational combination should satisfy at least one of five criteria: complementary spatial action, non-overlapping toxicity, oxygen or redox compensation, immune amplification, or resistance bypass [79,133].

Photothermal therapy is mechanistically attractive when heat improves tumor perfusion, disrupts membranes, enhances delivery or compensates for oxygen-limited ROS generation. Ferroptosis-inducing strategies are rational when PDT generates lipid peroxidation but tumor antioxidant systems limit death propagation. Chemotherapy or photoactivatable prodrugs are justified when light-controlled drug release improves spatial selectivity or when cytotoxic drugs target cells that survive oxidative stress. Hypoxia-activated prodrugs can be paired with PDT when treatment-induced oxygen depletion is used as a trigger rather than regarded only as a limitation. Radiotherapy may cooperate with PDT through spatial complementarity and oxidative stress amplification but requires careful dose scheduling and normal-tissue assessment.

Checkpoint blockade is rational only when PDT measurably enhances antigen release, dendritic-cell priming or T-cell infiltration. For BODIPY, the key advantage is that several combination functions can be built into the same molecular or supramolecular logic. A BODIPY scaffold may provide fluorescence guidance, red/NIR light absorption, Type I or Type II ROS generation, organelle targeting and activatable behavior. However, this modularity should be used sparingly. Every added module increases synthetic burden, formulation heterogeneity, pharmacokinetic uncertainty and regulatory complexity. The strongest BODIPY combinations will be mechanistically minimal: one photosensitizer, one clearly defined biological limitation and one partner intervention that addresses that limitation [134].

### 5.4. Translational Filters for Serious Candidates

The BODIPY PDT field now requires stronger preclinical discipline. Many candidates are evaluated under conditions that do not predict clinical performance: high light doses, normoxic monolayer cultures, non-standard ROS probes, short follow-up, poorly reported fluence rates, limited pharmacokinetics and subcutaneous tumor models used to support broad claims. Such studies can identify promising chemistry, but they are insufficient for translational prioritization.

A serious BODIPY PDT candidate should pass several filters. First, ROS identity must be defined using orthogonal assays. Singlet oxygen, superoxide, hydroxyl radicals and lipid peroxides should not be inferred from a single non-specific dye. Second, photodynamic output should be measured in the final biological formulation, not only in organic solvent. Aggregation, protein binding and nanocarrier encapsulation can alter fluorescence, triplet formation and ROS yield. Third, photobleaching, dark toxicity, hemocompatibility, long-term phototoxicity and normal-tissue retention should be reported. Fourth, pharmacokinetics, biodistribution and clearance must be matched to the proposed indication. A topical or intraoperative agent has different requirements from a systemically administered nanotheranostic. Fifth, light dose, fluence rate, wavelength, tissue optical assumptions and device geometry must be stated with enough detail to allow reproduction [135].

As summarized in Table 2, many representative BODIPY systems still lack complete PK, biodistribution, clearance and long-term safety evidence. Model selection should also match the biological claim. Immune-competent and orthotopic models are required when systemic immunity, abscopal effects or checkpoint synergy are proposed. Claims of oxygen-adaptive performance require oxygen mapping or hypoxia markers and testing under relevant oxygen conditions. Organellar targeting claims require co-localization, perturbation of organelle function and evidence that localization changes the death mechanism. These filters should not be viewed as obstacles. They are the basis for distinguishing clinically plausible BODIPY PDT from elegant but non-translatable photochemistry [136].

### 5.5. Translational Minimalism

The most clinically credible BODIPY systems may not be the most complex. Translation favors manufacturable chemistry, stable formulation, scalable quality control, predictable pharmacology, realistic illumination and a disease-site logic that clinicians can implement. This is particularly important because PDT is a drug–device therapy. A photosensitizer cannot be judged independently of how light will be delivered, how deeply it must act, how oxygenated the lesion is and whether treatment can be integrated into surgical, endoscopic or outpatient workflows [137].

Translational minimalism does not mean low ambition. It means matching complexity to need. A superficial basal-cell lesion may require a simple, photostable, red-light-responsive formulation rather than an elaborate oxygen-generating nanoplatform. An intraoperative margin application may require image contrast, rapid clearance and precise activation more than deep-tissue penetration. A hypoxic solid tumor application may justify Type I bias or hypoxia-activated combination logic, but only if illumination can be delivered and the formulation is reproducible. A vaccine-like strategy may justify immune adjuvant coupling, but only if immune priming is demonstrated [138].

This perspective should reshape how BODIPY photosensitizers are prioritized. Instead of asking which molecule gives the highest ROS signal in vitro, the field should ask which system has the clearest route to a clinical problem: what lesion, what light source, what formulation, what biological vulnerability and what measurable endpoint? BODIPY’s value lies in its programmability, but translation will depend on restraint [139].

### 5.6. Practical Roadmap

A practical roadmap for new BODIPY-photosensitizer-based PDT should begin with indication, not molecules. The first step is to choose a disease setting in which PDT has a credible role: superficial cancer, premalignant mucosal disease, endoscopic lesions, intraoperative margins, residual local disease, or selected image-guided interstitial applications. The second step is to define light access: external illumination, endoscopy, optical fiber, interstitial diffuser, intraoperative field or wearable device. The third step is to map the relevant oxygen and redox environment, because this determines whether Type II singlet oxygen, Type I radicals, oxygen supply, oxygen sparing or hypoxia exploitation should be prioritized. The fourth step is to select the BODIPY scaffold modification: wavelength tuning, triplet-state engineering, aggregation control, targeting, activatable logic or combination coupling [60].

Only after these steps should biological validation begin. ROS identity should be measured under relevant oxygen levels; localization should be linked to cell death mechanism; immune effects should be tested in immune-competent models; and formulation should be simplified rather than expanded. Finally, the candidate should be matched to deployable light hardware and evaluated with transparent dosimetry, pharmacokinetics, biodistribution, dark toxicity and normal-tissue safety.

The future of BODIPY PDT is therefore not the pursuit of universally stronger photosensitizers. It is the development of indication-first, oxygen-aware and image-guided systems designed for biological selectivity in which BODIPY chemistry is deliberately aligned with tumor anatomy, microenvironmental state, therapeutic mechanism and clinical workflow.

## Figures and Tables

**Table 1:** Class-level comparison of photosensitizer platforms.

Class	Optical/ROS Characteristics	Chemical Tunability	Imaging Compatibility	Major Strengths	Major Limitations	Clinical Maturity
Porphyrins [46,60]	Intense higher-energy band with weaker red bands; established triplet photochemistry, commonly Type-II-dominant	Peripheral substitution, metallation, conjugation and prodrug/formulation strategies	Fluorescence can support photodynamic diagnosis and guidance but remains compound- and environment-dependent	Established clinical PDT precedent and mature light-delivery/dosimetry workflows	Aggregation, heterogeneous distribution, prolonged photosensitivity and suboptimal long-wavelength absorption for some early agents	Clinically established for multiple agents or PpIX-generating prodrugs; indication and jurisdiction vary
Chlorins [46,60]	Strong red-region absorption relative to corresponding porphyrin patterns; efficient triplet formation, commonly Type-II-dominant	Peripheral functionalization, metal insertion and conjugation; highly functionalized derivatives can complicate regioisomer control	Many retain fluorescence suitable for image-guided PDT	Red-light efficiency combined with clinical PDT precedent	Hydrophobicity and aggregation, photobleaching/degradation, formulation needs and oxygen dependence	Clinically established for selected agents and indications
Phthalocyanines [60,65]	Intense red/far-red Q-band absorption; efficient triplet/Type II chemistry in suitable derivatives; mixed ROS/thermal behavior is possible	Central metal, peripheral substituents and axial ligands provide broad control	Fluorescence is possible but can be reduced by aggregation or triplet/thermal routing	Strong red/far-red absorption and extensive metal/substituent design space	Pronounced aggregation and aqueous insolubility; formulation can determine photophysics, PK and manufacturability	Intermediate and jurisdiction-dependent; less broadly standardized than established porphyrin/chlorin PDT
Cyanine dyes [66,67]	Red/NIR absorption and emission are readily accessible; native ISC/ROS can be weak, and photothermal pathways are common	Polymethine length, end groups, meso substitution, charge and appended ligands are tunable	Strong NIR fluorescence-imaging compatibility; imaging authorization does not establish a PDT indication	Clinically mature NIR imaging and a natural route to image-guided or activatable phototheranostics	Photobleaching/photooxidation, aggregation/protein binding and often insufficient native ROS generation	High for optical imaging, but low/emerging for PDT
AIE-based photosensitizers [55]	Aggregate-state emission and structure-dependent Type I/II ROS; no single fixed chromophore or spectral window	Broad donor–acceptor, rotor, charge, targeting and supramolecular design space	Strong aggregate-state fluorescence and imaging contrast	Functional emission and photosensitization in the assembled state; supports image-guided multimodal systems	Morphology-dependent performance; formulation identity, reproducibility, clearance and regulatory characterization are demanding	Predominantly preclinical/emerging for PDT
BODIPYs [27,30,59]	Parent dyes are visible-absorbing and fluorescence-favored; red/NIR absorption and Type I/II or thermal pathways require deliberate engineering	Multiple addressable scaffold positions enable coordinated tuning of spectrum, ISC/ROS, solubility, targeting, activation and assembly	Strong fluorescence and narrow bands; imaging must be balanced against triplet/thermal energy routing	Structural programmability and multifunctional co-design within one molecular or supramolecular framework	Not intrinsically long-wavelength or ROS-efficient; aggregation, formulation, coupled photophysical trade-offs and limited clinical pharmacology remain important	Preclinical/emerging as a PDT class

Note. Entries describe recurring class-level tendencies rather than properties of every derivative. Clinical maturity is time- and jurisdiction-dependent; authorization for optical imaging is not equivalent to a PDT indication.

**Table 2:** Representative BODIPY-based photoactive systems and their evidence maturity.

System/Reference	Design Strategy	Optical/Irradiation Conditions	ROS Mechanism and Photostability	Phototoxicity/Quantitative Benchmark	In Vivo Performance	PK/Biodistribution/Safety	Key Limitation
2I-BDP [73]	Direct 2,6-diiodination; internal heavy-atom-enhanced ISC	λ_abs_ 534 nm (MeOH); cells: 535 ± 25 nm, 5 mW cm^−2^, 1 min	Type II ^1^O_2_ (1268 nm emission and DPBF). More photobleaching-resistant than Rose Bengal under repeated 546 nm, 0.1 W irradiation; numerical loss NR	^1^O_2_ luminescence 1.34-fold that of Rose Bengal under the same MeOH/514 nm/100 mW assay; not an absolute Φ_Δ_. HeLa light-dependent death at 1 μM; IC_50_ and PI NR	NR	PK, biodistribution and systemic safety NR; no toxicity at 1 μM in the qualitative dark cell assay	Green-light, cell-only study without dose-response or translational evidence
BDP-5 NPs [75]	Heavy-atom-free twisted donor-phenyl-BODIPY; F127 formulation	Aqueous ROS assay: 504-nm excitation; cells/mice: green LED, 20 mW cm^−2^, 20 min; exact treatment wavelength NR	Type II ^1^O_2_; intracellular total ROS detected with DCFH-DA. Photostability NR	ΔE_ST ≈ 0.44 eV; numerical Φ_Δ_, dark/light IC_50_ and PI NR	HeLa tumor-bearing mice received directly administered nanoparticles; tumor size and H&E/TUNEL were evaluated over 3 weeks; quantitative BDP-5 outcome NR	PK, systemic biodistribution, normal-organ safety and blood safety NR	Direct local administration and green light; quantitative efficacy and systemic evidence incomplete
BDP-6@F127 NPs [83]	Heavy-atom-free formylated NIR aza-BODIPY in F127; combined PDT/PTT	λ_abs_ 772 nm (DMSO); cells/mice: 808 nm, 1.5 W cm^−2^, 5 min; mice irradiated 24 h after i.v. dosing	Type I O_2_•^−^/•OH plus Type II ^1^O_2_, with photothermal conversion. Photothermal performance retained over five heating–cooling cycles; photostability tested over six 808 nm cycles, with numerical absorbance loss NR	Φ_Δ_ = 0.304% (DPBF versus ICG, 808 nm); at 30 μM, 79% and 68% Cal-33 killing under 20% and 1% O_2_, respectively	Cal-33 xenografts: complete growth inhibition for 14 days. Bilateral SCC-7 model: distant-tumor inhibition required anti-PD-1	Tumor fluorescence by 6 h, peak at 24 h and retained at 48 h; liver had the highest ex-vivo signal. Quantitative PK NR. No significant short-term body-weight, blood/biochemistry or major-organ H&E changes	Cy5.5-labeled biodistribution is not molecular PK; short follow-up and high irradiance/thermal contribution
Glu-BDP2 [85]	GGT-responsive γ-glutamyl cage; heavy-atom-free SOCT-ISC, mitochondrial targeting and mitophagy-linked fluorescence feedback	Parent BDP2: λ_abs_ 660 nm; A549 cells: 660 nm, 20 mW cm^−2^, 20 min. Glu-BDP2-specific spectrum and irradiation protocol NR	SOCT-ISC platform with Type II ^1^O_2_; Glu-BDP2-specific quantitative activation/ROS data NR. Photostability NR	Glu-BDP2 Φ_Δ_, IC_50_ and PI NR	Implanted-tumor ablation without healthy-tissue damage was reported; tumor model, protocol and numerical outcome NR	PK and biodistribution NR; safety limited to a qualitative absence of healthy-tissue damage	Recoverable quantitative evidence mainly concerns parent BDP2; Glu-BDP2 translational characterization is incomplete
BDP 1 [86]	*N*,*N*-Diethylaminomethylphenyl aza-BODIPY; pH activation and lysosomal targeting	λ_abs_ 680 nm; mouse exposure 54 J cm^−2^; treatment wavelength, irradiance and time NR	Type II ^1^O_2_. Photostability NR	Dark IC_50_ > 50 μM (cell line NR); light IC_50_, PI and numerical Φ_Δ_ NR	Tumor-bearing mice: 2 mg kg^−1^ and one PDT treatment strongly inhibited growth and could ablate tumors; model and numerical outcome NR	Organ distribution assessed qualitatively by fluorescence; PK NR; no body-weight loss reported	Cell/tumor identities, complete dosimetry, quantitative biodistribution and systemic safety NR
LipoHPM [87]	Acid-sensitive biotin-PEG/MBA BODIPY micelles plus hydralazine in pH-sensitive liposomes; lysosomal de-aggregation and penetration/metastasis module	NIR-emissive; λ_abs_ and treatment wavelength NR; mice: 100 mW cm^−2^, 20 min at 12 h after i.v. dosing	Type I and Type II ROS after disassembly/de-aggregation. Photostability NR	Quantitative ROS metric, dark/light IC_50_ and PI NR	4T1/Luci4T1 breast tumors in nude mice (*n* = 6/group); antitumor and antimetastasis effects reported; numerical response NR	Qualitative fluorescence tumor accumulation; PK NR. Body weight was monitored, but the result and systemic safety endpoints were NR	Multicomponent formulation; quantitative dosimetry, efficacy, PK/clearance and safety incomplete
B5@HMON [88]	Bithiophene aza-BODIPY in disulfide-containing HMON; GSH-responsive release/depletion and combined photoimmunotherapy	808 nm laser; λ_abs_, irradiance and duration NR	Combined PDT/PTT; Type I/II assignment and quantitative ROS metric NR. Photostability NR	B5 loading 11% (formulation metric); phototoxic IC_50_ and PI NR	4T1-bearing BALB/c mice; almost all tumors disappeared by day 14; denominator and longer follow-up NR; immune readouts assessed	PK and quantitative biodistribution NR; quantitative systemic safety and degradation endpoints NR	Carrier-dependent, multimechanism system without complete dosimetry or separation of PDT, PTT and immune contributions
Compound **1b** [89]	Mono-iodinated, fused/π-extended isoindole-BODIPY designed for red absorption, triplet access and heat generation	λ_abs_ 630 nm (water); cells: 630 nm, 2.00 mW cm^−2^, 40 min (5.0 J cm^−2^)	Predominantly oxygen-independent photothermal action under cellular treatment conditions. Φ_Δ_ = 0.26 ± 0.05 in MeOH-*d*_4_, but cellular ROS signals were small; activity at 0.2% O_2_ is not evidence of hypoxic Type I/II PDT. Photobleaching quantum yield Φ_d_ = (6.5 ± 0.6) × 10^−6^ in DMF under 630 nm irradiation	HeLa: light IC_50_ 0.0060 ± 0.0003 μM, dark IC_50_ > 5000 μM, PI > 830,000. At 0.2% O_2_: 0.0069 ± 0.0003 μM, >2500 μM and >360,000, respectively	NR	PK, biodistribution and systemic safety NR. Illuminated non-cancerous MRC-5 cells were also sensitive (IC_50_ 0.0126 ± 0.0006 μM)	No in vivo validation or tumor selectivity; 630 nm excitation; tissue-scale thermal dosimetry NR

Note. These values are study-specific benchmarks and must not be interpreted as a rank order. ROS assay, solvent/formulation, reference standard, oxygen condition, light wavelength and dose, sensitizer exposure and wash schedule, biological model and endpoint differ across studies; Φ_Δ_, IC_50_, PI, photostability and tumor outcomes are directly comparable only under matched conditions. NR, not reported; ISC, intersystem crossing; PI, phototoxicity index; PK, pharmacokinetics; PDT, photodynamic therapy; PTT, photothermal therapy; SOCT-ISC, spin–orbit charge-transfer intersystem crossing.

## Data Availability

No new data were created or analyzed in this study. Data sharing is not applicable.

## Abbreviations

The following abbreviations are used in this manuscript:

ACQ	Aggregation-caused quenching
ATP	Adenosine triphosphate
BODIPY	Boron–dipyrromethene
CD8	Cluster of differentiation 8
CT	Charge transfer
DAMP	Damage-associated molecular pattern
ER	Endoplasmic reticulum
GPX4	Glutathione peroxidase 4
GSH	Glutathione
HIF	Hypoxia-inducible factor
HMGB1	High-mobility group box 1
HOMO-LUMO	Highest occupied molecular orbital-lowest unoccupied molecular orbital
ICB	Immune checkpoint blockade
ICD	Immunogenic cell death
ISC	Intersystem crossing
NIR	Near-infrared
NIR-II	Second near-infrared window
OLED	Organic light-emitting diode
PDT	Photodynamic therapy
PEG	Polyethylene glycol
PK/PD	Pharmacokinetics and pharmacodynamics
RNS	Reactive nitrogen species
ROS	Reactive oxygen species
S_0_	Ground singlet state
S_1_	First excited singlet state
T_1_	First excited triplet state
TME	Tumor microenvironment
